# Machine Learning to Identify Genetic Salt-Losing Tubulopathies in Hypokalemic Patients

**DOI:** 10.1016/j.ekir.2022.12.008

**Published:** 2022-12-24

**Authors:** Elizabeth R. Wan, Daniela Iancu, Emma Ashton, Keith Siew, Barian Mohidin, Chih-Chien Sung, China Nagano, Detlef Bockenhauer, Shih-Hua Lin, Kandai Nozu, Stephen B. Walsh

**Affiliations:** 1 Department of Renal Medicine, University College London, London, UK; 2 North East Thames Regional Genetics Service Laboratories, Great Ormond Street Hospital for Children National Health Service Foundation Trust, London, UK; 3 Division of Nephrology, Department of Medicine, Tri-Service General Hospital, National Defense Medical Center, Taipei, Taiwan; 4 Department of Pediatrics, Kobe University Graduate School of Medicine, Kobe, Japan; 5 Department of Pediatric Nephrology, Great Ormond Street Hospital for Children National Health Service Foundation Trust, London, UK

**Keywords:** diagnosis, genotyping, machine learning, tubulopathy

## Abstract

**Introduction:**

Clinically distinguishing patients with the inherited salt-losing tubulopathies (SLTs), Gitelman or Bartter syndrome (GS or BS) from other causes of hypokalemia (LK) patients is difficult, and genotyping is costly. We decided to identify clinical characteristics that differentiate SLTs from LK.

**Methods:**

A total of 66 hypokalemic patients with possible SLTs were recruited to a prospective observational cohort study at the University College London Renal Tubular Clinic, London. All patients were genotyped for pathogenic variants in genes which cause SLTs; 39 patients had pathogenic variants in genes causing SLTs. We obtained similar data sets from cohorts in Taipei and Kobe, as follows: the combined data set comprised 419 patients; 291 had genetically confirmed SLT. London and Taipei data sets were combined to train machine learning (ML) algorithms, which were then tested on the Kobe data set.

**Results:**

Single biochemical variables (e.g., plasma renin) were significantly, but inconsistently, different between SLTs and LK in all cohorts. A decision table algorithm using serum bicarbonate and urinary sodium excretion (FE_Na_) achieved a classification accuracy of 74%. This was superior to all the single biochemical variables identified previously.

**Conclusion:**

ML algorithms can differentiate true SLT in the context of a specialist clinic with some accuracy. However, based on routine biochemistry, the accuracy is insufficient to make genotyping redundant.

## Graphical abstract

**Figure:**
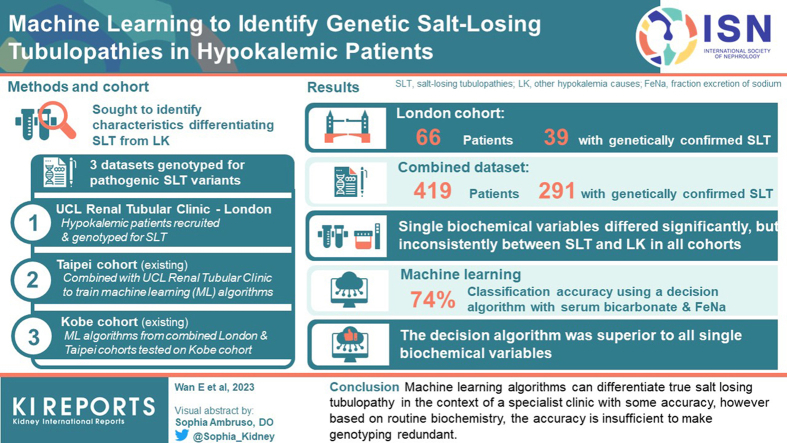

Chronic hypokalemia because of renal wasting, associated with a metabolic alkalosis, is a frequent presentation of GS (OMIM 263800, here referred to as GS) and classic, type 3, BS (607364, BS3), which are hereditary SLTs. GS is caused by inactivating mutations of *SLC12A3*, which encodes the apical thiazide-sensitive sodium chloride cotransporter of the distal convoluted tubule.^1^ It characteristically causes hypokalemic metabolic alkalosis with hypomagnesemia and hypocalciuria, although hypomagnesemia may be absent and the hypocalciuria is very variable.^2^ It is an autosomal recessive disease with an estimated mutant gene frequency of between 1% to 3.6%.^3^ Its overall prevalence is approximately 10 of 40000, making it the most common inherited tubulopathy.^4^ BS is rarer but also characterized by hypokalemic metabolic alkalosis. This is due to underactivity of the bumetanide-sensitive sodium potassium chloride cotransporter, NKCC2, present in the thick ascending limb of the loop of Henle. Type 3 BS (BS3) is the commonest form and is caused by variants in *CLCNKB*, which encodes a basolateral chloride channel, CLCKb.^5^ Like GS, it is often diagnosed following the incidental discovery of hypokalemia and alkalosis during adulthood. Although the BS complex tends to cause hypercalciuria and not hypomagnesemia, BS3 has a variable phenotype that may be indistinguishable from GS. This is probably because of the distribution of CLCKb, which is present both in the thick ascending limb and distal convoluted tubule.^6^ BS1, 2, and 4 are associated with severe salt and water loss and failure to thrive during infancy and are rarely diagnosed in adults.

The clinical diagnosis of GS and BS is made more difficult by conditions which mimic their biochemical phenotype (a hypokalemic metabolic alkalosis). In children, it may be mimicked by inherited conditions such as congenital chloride diarrhea or cystic fibrosis. In adults, it can be mimicked by vomiting, laxative abuse, or surreptitious diuretic use.^7^ Together, these purging behaviors have been called “Pseudo-Bartter” syndrome, and in some series comprise over 50% of patients being investigated for hypokalemia.^8^ Because they are almost always covert and indicative of an often-undiagnosed eating disorder, they can be difficult to distinguish from either GS or BS without diagnostic genetic testing.

However, it is important to diagnose the “pseudo-Bartter” purging behaviors. Purging patients are likely to take up time and resources in a clinical environment (a nephrology clinic) that is inappropriate and unhelpful for them. Further, diagnosing a purging behavior is significant because it is likely to be a presentation of bulimia nervosa, or another eating disorder. Patients with bulimia have an all-cause mortality of up to 8 times that of the general population^9^ and 3 times greater than that for bipolar disorder and depression.^10^ An early diagnosis and referral to appropriate psychiatric services may be lifesaving.

It is therefore unsurprising that the kidney disease: improving global outcomes working group on GS recommends genetic testing for diagnosis.^11^ However, even now, access to genetic testing is unequal in centers across the UK, and the disparity is greater in many other countries. Here we present a multicenter, international study of those clinical factors which differ between those with genetic and other causes of hypokalemia. We describe a well characterized local cohort of patients who were investigated for possible SLT by genetic and biochemical testing. We combine these data with 2 other similar data sets and apply statistical and ML approaches to identify strategies to predict which patients will have biallelic variants causing GS or BS.

## Methods

The University College London Department of Renal Medicine runs a specialist clinic for renal tubular disorders. We carried out a prospective, observational cohort study of patients referred to the University College London tubular clinic (the “London cohort”) between 2012 and 2017. The study was approved under the ethical approval “Identification of genes involved in renal, electrolyte, and urinary tract disorders” (REC reference 05/Q0508/6).

We enrolled all patients who were persistently hypokalemic at point of referral, requiring potassium supplementation, and who consented to genotyping. Patients were excluded if they were hypertensive, had chronic kidney disease stage 4 or 5, incomplete laboratory or clinical data, or other causes for their hypokalemia (e.g., familial hypokalemic paralysis), or were not genotyped.

All London cohort patients were genotyped using the Multiplicom TUBMASTR^12^ panel by multiplex polymerase chain reaction and next generation sequencing, for a panel of genes associated with inherited tubular disease, including *SLC12A3, CLCNKB, HNF1B, SLC12A1, KCNJ1, and BSND*.^13^

Data contemporaneous to the first clinic visit were collected from the electronic patient record. This included data for age, gender, blood pressure, serum and urinary biochemistry, as measured in the Royal Free Hospital biochemistry laboratory by standard methods. From this, the urinary fractional excretion (FE) of sodium, chloride, and magnesium was calculated. The London cohort comprised 66 genotyped patients, with 31 datapoints per patient. Of these, 39 had genetically confirmed SLT.

These data were analyzed, and descriptive statistics (including *t*-tests, Mann-Whitney *U* tests, and receiver operator characteristic [ROC] curves) were generated using Graphpad PRISM (GraphPad Software, La Jolla, CA) version 8.0 and the open access statistics package RStudio (RStudio, PBC, Boston, MA; https://www.rstudio.com), to identify variables that would be able to correctly classify patients as LK or SLT. Missing data were managed with multivariate imputation by chained equations (“mice” package, https://cran.r-project.org/web/packages/mice).

Because similar studies have been published in the last 5 years, we decided to incorporate these data. A data set from Taipei, Taiwan, published in 2017 (the “Taipei cohort”),^14^ comprised 87 patients, with 27 datapoints per patient. There were 43 genetically confirmed SLT patients in this cohort, who were selected in a very similar fashion to our own. A large Japanese cohort has also recently been published.^15^^,^^16^ This cohort (the “Kobe cohort”) was 266 patients strong. Each patient had 20 data points and there were 209 genotyped SLT patients. The London and Taiwan data sets exclusively included adult patients, whereas the Kobe data set included a mixture of children and adults. All biochemistry was taken at first attendance to clinic, that is, shortly after referral.

Because descriptive statistics had been published on these data sets already, we took the view that duplicating this would be redundant. We did, however, harmonize the data sets so that they all had the same 18 datapoints and reanalyzed the combined data set using the same methods as the London cohort alone.

In addition, we employed an approach using ML algorithms to see whether we could correctly classify SLT patients on the basis of known clinical and biochemical datapoints in the whole data set.

The London and Taipei cohorts were harmonized (to the common 18 datapoints present in all cohorts) and combined. This combined data set was used as a “training data set” to train a number of ML algorithms. The large total number of patients helps to reduce bias. We could not control for any bias on the part of those referring patients into these centralized tertiary clinics.

Then, classification algorithms were applied to all 18 datapoints in the training data set to classify the patients as LK or SLT. All of the algorithms were then rerun against the Kobe cohort, which comprised the “testing dataset.”

ML analyses were performed using the open access Waikato Environment for Knowledge Analysis, version 3.8.3, University of Waikato, New Zealand platform.

## Results

### London Cohort

The London cohort comprised 66 patients, ranging in age from 17 to 85 (mean age 41) years (Table S1). There were 38 female and 29 male patients.

Direct comparison between the SLT and LK groups shows significant differences for a number of variables. SLT patients tended to have lower serum potassium, magnesium, and higher serum renin concentrations. SLT patients had significantly greater polyuria, as evidenced by a lower urinary creatinine concentration. They had a higher FE of chloride and FE of sodium.

**Figure 1:**
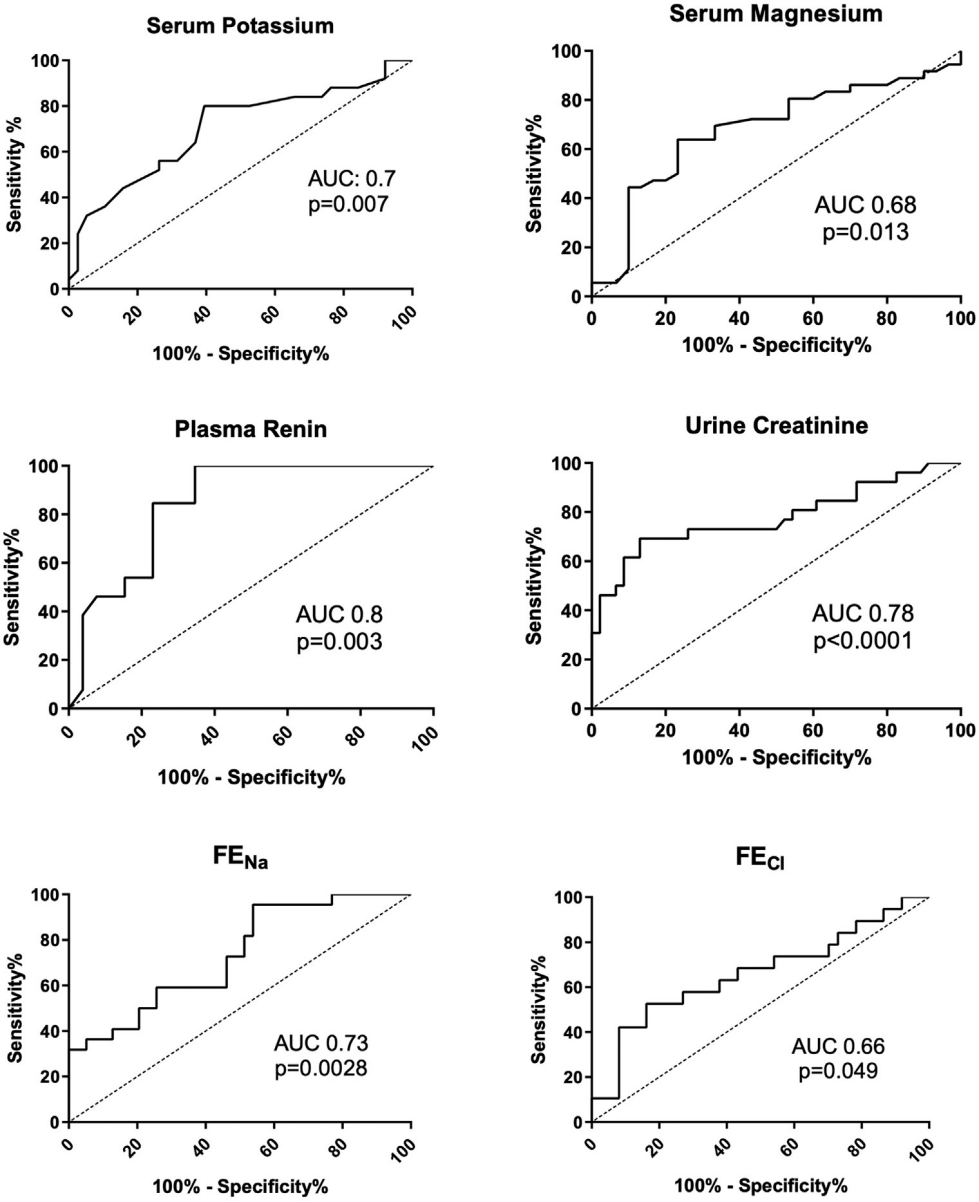
Receiver operator characteristic curves of significantly different biochemical values between SLT and LK in the London cohort. AUC and statistical significance (*P* value) indicated for each curve. AUC, area under the curve; FE, fractional excretion.

ROC curves were generated for the same data. The serum potassium was a fair discriminator of SLT versus LK (AUC 0.7 ± 0.07, *P* = 0.007). The plasma renin concentration was a good discriminator (AUC 0.8 ± 0.07, *P* = 0.003), as was the urinary creatinine concentration (AUC 0.8 ± 0.06, *P* < 0.0001) (see Figure 1).

### Combined Cohort

The combined data set (London, Taipei, and Kobe cohorts) was 419 patients, of which 291 had genotyped SLT. The median age was 26 (interquartile range 11−40) years, with a slight female preponderance (239 female and 180 male patients). There were significant differences between the SLT and LK groups. SLT patients presented at a younger age. The serum potassium and aldosterone concentrations were lower in the SLT group. The urinary FE of sodium, chloride, and magnesium were all significantly higher (all *P* < 0.0001) in the SLT than the LK group (see Table 1).

**Table 1:** Bivariate analysis of the combined cohort

Variable	LK (*n* = 128)	SLT (*n* = 291)	Test	*P*-value	Differences in medians (95% CI)
Age (yr)	35 (25–46)	19 (9–38)	MW	<0.0001	16 (9–16.9)
BMI	16.45 (14.9–18.3)	17 (15–21)	MW	Ns	−1.25 (−1.8 to 0)
Serum potassium (mmol/l)	2.8 (2.4–3.2)	2.7(2.3–2.9)	MW	0.0093	0.1 (0–0.3)
Serum bicarbonate (mmol/l)	30 (26.3–36.4)	30.3 (28–33)	MW	Ns	−0.3 (−1.1 to 1.2)
Serum magnesium (mmol/l)	0.92 (0.8–1.8)	1.4 (0.9–1.8)	MW	Ns	−0.48 (−0.26 to 0.05)
Serum creatinine (μmol/l)	77 (55–106)	45.9 (32.7–70.7)	MW	<0.0001	31 (21.2–34.9)
Renin (nmol/l/hr)	1.5 (0.7–1.5)	1.4 (0.8–2.4)	MW	Ns	0.1 (−0.3 to 0.2)
Aldosterone (pmol/l)	400 (186–1250)	225 (132–444)	MW	0.0001	175 (68–244)
Urinary sodium (mmol/l)	52.5 (21.5–126.5)	93.5 (5.25–127)	MW	0.0003	−41 (−43 to −13)
Urinary potassium (mmol/l)	39 (17.4–81.7)	41.15 (24.8–62.3)	MW	<0.0001	−59 (−59 to −29)
Urinary creatinine (mmol/l)	13.88 (8.5–29.5)	17 (6.5–63.5)	MW	Ns	−3.21 (−8.25 to 1.99)
Urinary magnesium (mmol/l)	3.6 (1.7–7)	2.35 (1.4–3.7)	MW	0.0015	1.25 (0.41–2.3)
Fractional excretion of chloride (%)	0.36 (0.17–1.17)	1.007 (0.64–1.62)	MW	<0.0001	−0.64 (−0.66 to −0.3)
Fractional excretion of sodium (%)	0.39 (0.07–0.8)	0.6 (0.39–0.92)	MW	<0.0001	−0.21 (−0.33 to −0.13)
Fractional excretion of magnesium (%)	0.42 (0.14–0.9)	1.2 (0.26–2.92)	MW	<0.0001	−0.78 (−1.09 to −0.14)

BMI, body mass index; CI, confidence interval; LK, hypokalemia; MW, Mann-Whitney; SLT, salt-losing tubulopathy.

Other LK vs. SLT patients values displayed are median with interquartile range. Test shows which statistical test was used to determine significance, MW for non-normally distributed data or the *t* test for normally distributed data.

**Figure 2:**
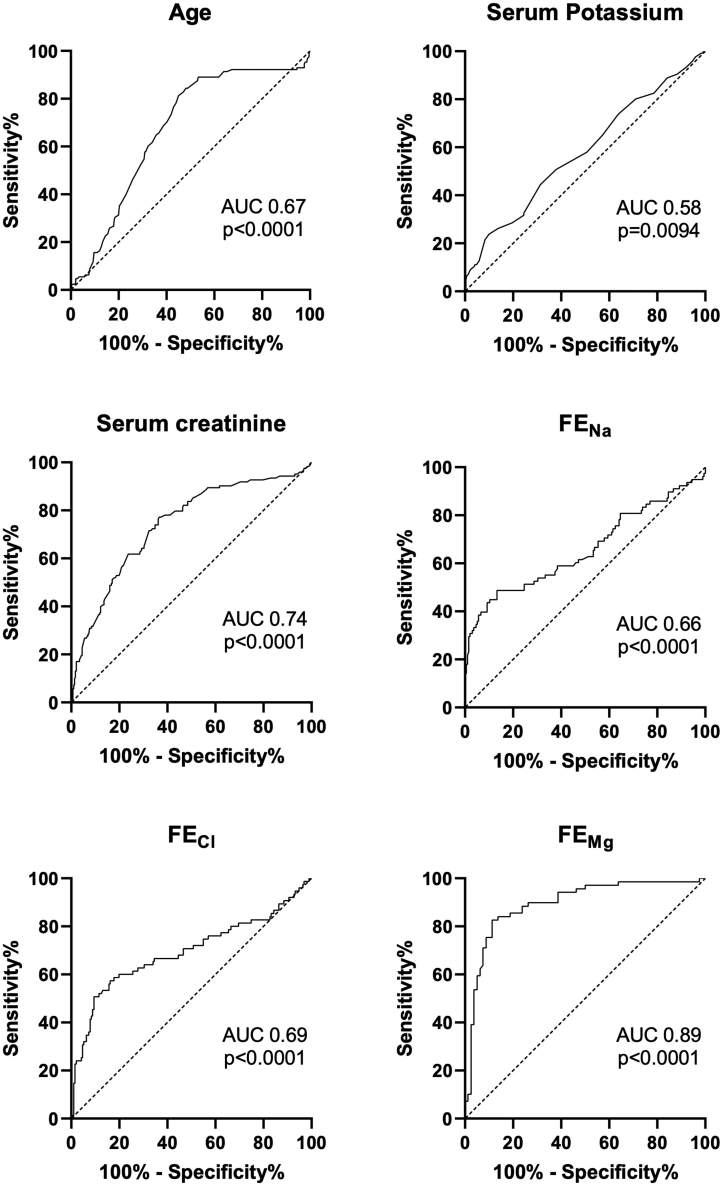
Receiver operator characteristic curves of significantly different biochemical values between SLT and LK in the combined cohort. AUC and statistical significance (*P* value) indicated for each curve. AUC, area under the curve; FE, fractional excretion.

Generation of ROC curves showed that the urinary FE of chloride was a fair discriminator of SLT versus LK (AUC 0.69 ± 0.04, *P* < 0.0001) and the FE of magnesium was a good discriminator (AUC 0.89 ± 0.03, *P* < 0.0001). (Figure 2).

### ML

Classification algorithms were run against the training set (London and Taipei cohorts), using 10-fold cross validation. These were then run against the testing data set (Kobe cohort).

Intriguingly, the 2 algorithms with the highest accuracy in the testing set used simple rules to predict LK or SLT. These were a decision table and a “decision stump” (an abbreviated decision tree with only 1 branch) algorithm. The better performing decision table (predictive accuracy in the training set 77% and 75% in the testing set) used a combination of the serum bicarbonate and the FE_Na_. The decision stump (predictive accuracy 66% and 61%) simply discriminated based on the FE of chloride (FE_Cl_). These attributes were all predicted in the attribute selection step. These simple models are detailed in Supplementary Table S2.

We compared these 2 algorithms to the single biochemical variables identified by conventional statistical methods as the 2 best discriminators in the London and combined cohorts. We did this by running each of these variables (FE_Mg_, renin, serum creatinine, urine creatinine) as a decision stump algorithm trained on the London and Taipei cohorts and tested on the Kobe cohort. The ML algorithms were superior discriminators than the variables identified by standard statistical methods in the London or combined cohorts (Table S3), despite an apparently superior ROC AUC value for FE_Mg_ in the combined cohort.

As a secondary analysis, we combined all of the cohorts into 1 harmonized data set and retrained algorithms using 10-fold cross validation, using either the best performing 8 datapoints or all 18. This resulted in higher predictive accuracy, the best performing algorithm being a Random Forest (predictive accuracy 81.9%, ROC AUC 0.89).

### Subgroup Analyses

**Figure 3:**
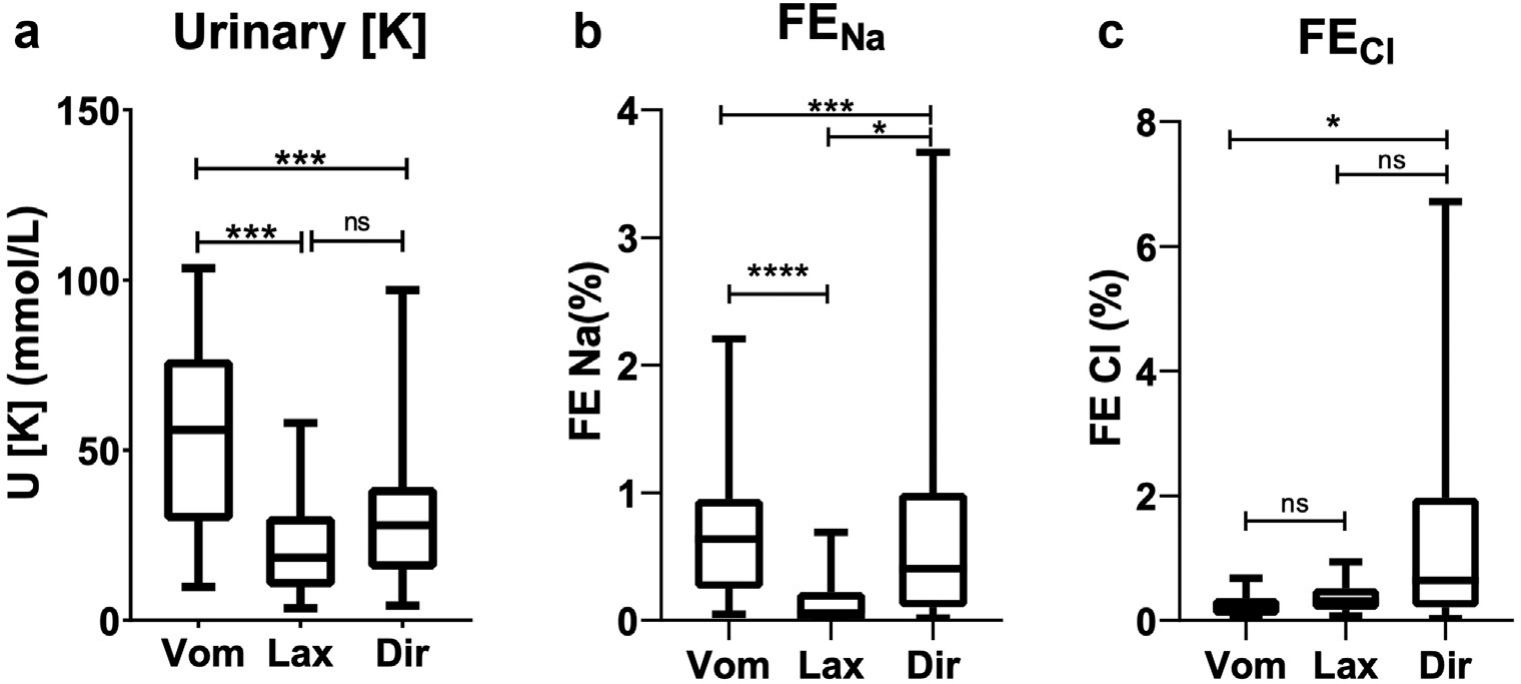
Significant biochemical differences between LK purging subgroups; vomiters (Vom), laxative abusers (Lax) and diuretic abusers (Dir). Significance is determined by the Mann-Whitney test (between 2 groups) or the Krukshal-Wallis test (between all groups). ns, nonsignificant; ∗, *P* < 0.05, ∗∗∗, *P* < 0.001, ∗∗∗∗, *P* < 0.0001. FE, fractional excretion.

LK patients who had been assigned a diagnosis of vomiting or diuretic or laxative abuse (only the Taipei and Kobe cohorts) were compared. The most significant differences between the 3 groups were the urinary potassium, FE_Na,_ and FE_Cl,_ which are shown in Figure 3.

Among the SLT patients, we compared the GS patients to those with BS type 3. The most significant differences were in the serum bicarbonate, renin activity, aldosterone, urine creatinine, FE_Cl,_ and FE_Mg_. These are shown in Supplementary Figure S1.

### Genetic Analysis

The genotyping results for SLT patients from the combined cohort are presented in Supplemental Table S4. There were 291 SLT patients, of which pathologic variants were present in *SLC12A3* (235), *CLCNKB* (52), *SLC12A1* (2) and *KCNJ1* (2).

## Discussion

Inherited salt-wasting disorders are uncommon and can pose a diagnostic and therapeutic challenge.^17^ Presentation is typically with the, often incidental, finding of hypokalemia, in the setting of a variable metabolic alkalosis without hypertension.

In adults, differential diagnoses are unlikely to include other known genetic diseases with the exception of *HNF1B* variants, which in the early stages may phenocopy GS/BS3. Disorders such as congenital chloride diarrhea and cystic fibrosis present in infancy and are not viable differential diagnoses in adults.

Therefore, the main differential diagnoses in adults comprise patients with eating disorders engaging in purging behaviors like vomiting, surreptitious diuretic, or laxative use.^8^ The eating disorders associated with purging are prevalent; anorexia nervosa has a lifetime prevalence of up to 2.2%^18^; bulimia has a lifetime prevalence of 1%.^19^ The prevalence of purging behavior within these eating disorders is as high as 86%.^20^ This is a much higher prevalence than GS, the most prevalent of the SLTs.

Biochemical clues to patients engaging in purging behavior have been described. Prolonged vomiting will cause a metabolic alkalosis, which may be very severe^21^^,^^22^; indeed it has been reported that a serum bicarbonate of >45 mmol/L is almost always because of a gastric cause.^23^ Hypokalemia is due to urinary wasting (from both hyperaldosteronism [from volume contraction] and bicarbonaturia [from acid loss]; the latter may be more important because hypokalemia may be ameliorated by proton pump inhibition^24^). Avid chloride retention typically results in undetectably low urinary chloride concentrations.

Chronic diarrhea in laxative abusers causes an alkalosis in part from volume depletion and hyperaldosteronism; this is rarely severe; serum bicarbonate is rarely >34mmol/l.^25^ Potassium losses are intestinal, and urinary potassium and chloride concentrations are low. Surreptitious diuretic use will cause a hypokalemic metabolic alkalosis. However, there will be a chloruresis while the drug is working; this will resolve when the drug effect ceases. This variability in the urinary chloride concentration may help to make the diagnosis,^26^ which can be confirmed by detection of diuretic in the urine.^27^

However, differentiating individual purging behaviors may be difficult, or even futile. Purging patients are unlikely to admit these behaviors even on close questioning.^28^ Furthermore, purging patients rarely employ only 1 purging behavior; multiple purging behaviors are reported in as much as 52.5% of patients with eating disorders associated with purging.^20^ Therefore, we decided against trying to differentiate LK patients on the basis of their probable (or, occasionally proven) purging behavior, and treated them as 1 homogenous group in the London cohort, and in the overall analysis.

The 2017 consensus report from kidney disease: improving global outcomes recommends that “genetic testing should be offered to all patients with a clinical suspicion of Gitelman Syndrome (minimal criteria)”^11^. However, access to nationally accredited methods to make a genetic diagnosis of Bartter or GS are not universally available.

Moreover, genetic screening has some technical difficulties. Historically, apparent heterozygosity for SLT causing genes is common in patients presenting as SLT, as high as 15% to 20%^29^^,^^30^ However, the apparent lack of pathogenic variants on the other allele may have been due to large genomic rearrangements that are missed by direct sequencing and may account for up to 15% of the genetic defects responsible for autosomal recessive diseases.^31^

The frequency for heterozygote carriage of pathogenic variants in SLC12A3 is as high as 1% to 2.9%.^32^ SLC12A3 heterozygosity is often reported in patients presenting with hypokalemia, and as far as blood pressure goes they may have an intermediate phenotype between normal and GS.^33^ The significance of this observation is not known.

Because of the distribution of CLCKb (in the thick ascending limb and the early distal convoluted tubule), there is considerable overlap in the phenotype of Type 3 Bartter and GS, which may present identically.^34^^,^^35^ Seyberth proposed a different nomenclature to better reflect the correlation of the clinical features with the location in the nephron of the genetic lesion.^6^ For this reason, we considered all patients with GS or type 3 BS to be a homogeneous group, “SLT.” Phenotypic overlap also exists with *HNF1B* (which encodes the transcription factor HNF-1β); patients may present with typical electrolyte disturbances seen in GS, especially hypomagnesemia and hypocalciuria.^36^ Although to date, most centers offering genetic testing for GS would routinely also sequence *CLCNKB*, *HNF1B* is not normally also sequenced, although it was sequenced as part of the TUBMASTR panel used for the London cohort.

In this sense, the kidney disease: improving global outcomes recommendation that genetic screening should be a mandatory investigation for these patients is appropriate, especially if it is in the context of a panel of genes such as *HNF1B*, that include other diseases that may phenocopy GS. In the UK, the recent introduction of clinical exome sequencing makes the simultaneous screening of multiple candidate genes routine and makes the detection of variants in putative novel genes possible, at least in a research setting. One, rather obvious, limitation of our approach is that current routine genetic screening will not pick up patients who have pathogenic variants in putative novel genes that cause a salt-losing nephropathy.

In the London cohort, one of the best discriminators between SLT and LK patients is the plasma renin activity. Renin angiotensin aldosterone system activation is expected in SLT,^37^ but would be expected in purging patients also; it may be that intermittent volume depletion is a lesser renin stimulus than constant renal salt loss.

A raised FE_Cl_ has previously been reported in the SLT population^38^ and has been suggested as a part of clinical diagnostic criteria for GS.^11^ In our cohort, we observed a statistically significant difference between the FE_Cl_ and FE_Na_ in both groups (each higher in SLT than LK). We also found that the urinary creatinine concentration was significantly lower in SLT; we interpreted this as indicating greater polyuria in SLT compared to LK.

We found a lower serum potassium level in SLT compared to LK patients at their first clinic visit (difference in means 0.49, 95% confidence interval −0.817 to −0.153, *P* = 0.00479). This probably reflects the median difference between SLT patients with a constantly low serum potassium, compared to LK patients with a variable LK from purging.^39^

In the combined cohort, age at presentation was significantly younger in SLT than LK; this is probably because of the young age of presentation in SLT, particularly in the Kobe cohort.^15^ As in the London cohort, serum potassium in other cohorts was significantly lower in SLT, although this was a poor discriminator. The urinary FE of sodium, chloride and magnesium were all significantly increased in SLT, with the FE_Mg_ being the best discriminator.

The ML algorithms that performed best in both the training and testing datasets were a decision table that used the serum bicarbonate and FE_Na_ and a decision stump that only used the FE_Cl_. The decision table warrants some comment; it predicts SLT only when there is a relative natriuresis (FE_Na_ > 0.251%) and the serum bicarbonate is between 25.5 mmol/l and 38.5 mmol/l; whereas those with the same FE_Na_ and a more severe alkalosis (serum bicarbonate >38.5 mmol/l) were predicted as LK. Although very severe alkalosis has occasionally been reported in SLT, this is in the more severe infantile forms of BS^40^; in adults, severe alkalosis is most commonly associated with persistent vomiting.^21^^,^^22^ The relative naturesis in the same LK patients is counterintuitive; one would expect a low FE_Na_ in a vomiting patient. Yet in our subgroup analysis, the LK patients deemed to be vomiting had a median FE_Na_ of 0.64% (interquartile range 0.25−0.95%). This is difficult to explain unless there is more than 1 purging behavior, for example, diuretic abuse or using salt as an emetic.

The less accurate decision stump used only the FE_Cl_, in keeping with previous observations,^8^ in fact a recent series of SLT diagnosed in childhood reported a median FE_Cl_ of 1.62%.^41^ The combined cohort SLT patients had a median FE_Cl_ of 1.01% (interquartile range 0.64−1.67%) compared to LK who had a low median FE_Cl_ of 0.35% but a large interquartile range (0.17−1.17%), likely because of the contribution of diuretic-induced chloruresis in diuretic-abusing patients.

The predictive accuracy of these ML classification algorithms was better than the individual biochemical variables identified by standard statistical methods. Interestingly, it was also superior to that of expert clinicians; a recent report of the results of genotyping renal tubular disorders in adults in 3 European reference centers found a genetic confirmation rate of only 46% for GS.^13^

A limitation of this study is the background heterogeneity of the combined cohort. In particular, the Japanese cohort included pediatric patients, whereas London and Taipei focused solely on adult clinics. Though age is not a biologically plausible variable in serum biochemistry in these diseases, there are limited published data in this area. There is likely to be a degree of dietary variation among the different cohorts, which we did not have dietary data to explore.

## Conclusions

We present a new cohort of patients that have been investigated for SLT, all of whom have been comprehensively genotyped for genes known to cause or phenocopy SLT. We also present a combined data set of this and 2 other, recently published cohorts, to further interrogate the biochemical differences between SLT and LK patients. We present ML generated biochemical strategies to discriminate between SLT and LK, on the basis of the largest combined cohort of genotyped SLT patients described, to the best of our knowledge, in the literature. These data demonstrate the difficulties faced in trying to make a diagnosis based on biochemistry alone and underscores the necessity for comprehensive genetic diagnosis in this difficult set of patients.

## Disclosure

All the authors declared no competing interests.

## Supplementary Material

**Supplementary Methods.**

**Figure S1.** Significant biochemical differences between type 3 Bartter syndrome, Gitelman syndrome and other hypokalemic patients in the combined cohort. Data expressed as medians with 95% confidence interval. Significance is determined by the Mann-Whitney test (between 2 groups) or the Krukshal-Wallis test (between all groups). ns, nonsignificant; ∗*P* < 0.05; ∗∗∗*P* < 0.001; ∗∗∗∗*P* < 0.0001.

**Table S1.** Bivariate analysis of the London cohort.

**Table S2.** Comparison of the 2 best performing algorithms identified by ML.

**Table S3.** Decision table or stump rules.

**Table S4.** Genotyping data from all SLT patients.

**STROBE Statement.**
