# Supplementary material for: Machine Learning to Identify Genetic Salt-Losing Tubulopathies in Hypokalemic Patients
Source: Kidney Int Rep. 2022 Dec 24;8(3):556–65. doi: 10.1016/j.ekir.2022.12.008 (PMC10014379; doi:10.1016/j.ekir.2022.12.008)
Supplement: Supplementary File (PDF) [file mmc1.pdf]

Supplementary methods, supplementary data and STROBE statement

Supplementary Methods

ROC vs Predictive accuracy/Precision recall:

There is another factor to bear in mind when considering the different performances of the machine learning algorithms and the single variables selected by standard statistical methods. The combined dataset that we have used is highly skewed toward patients with genetic SLT, which comprise >50% of all of the cohorts in the dataset, and 67% of the combined dataset.

In such skewed datasets, a precision recall curve will often give a better idea of the performance of an algorithm than the ROC curve[41]. This may be a problem if the skewed nature of the dataset is unrepresentative of datasets that the algorithms will be used on. Obviously, this is not true in the general population and we would not argue that these algorithms are useful for population-based screening for SLTs. However, in hypokalaemic patients in whom a diagnosis of SLT is queried, the proportion of actual SLT is likely to be very high, and thus our datasets are likely representative of the relevant clinical scenarios.

<u>Supplementary data</u>

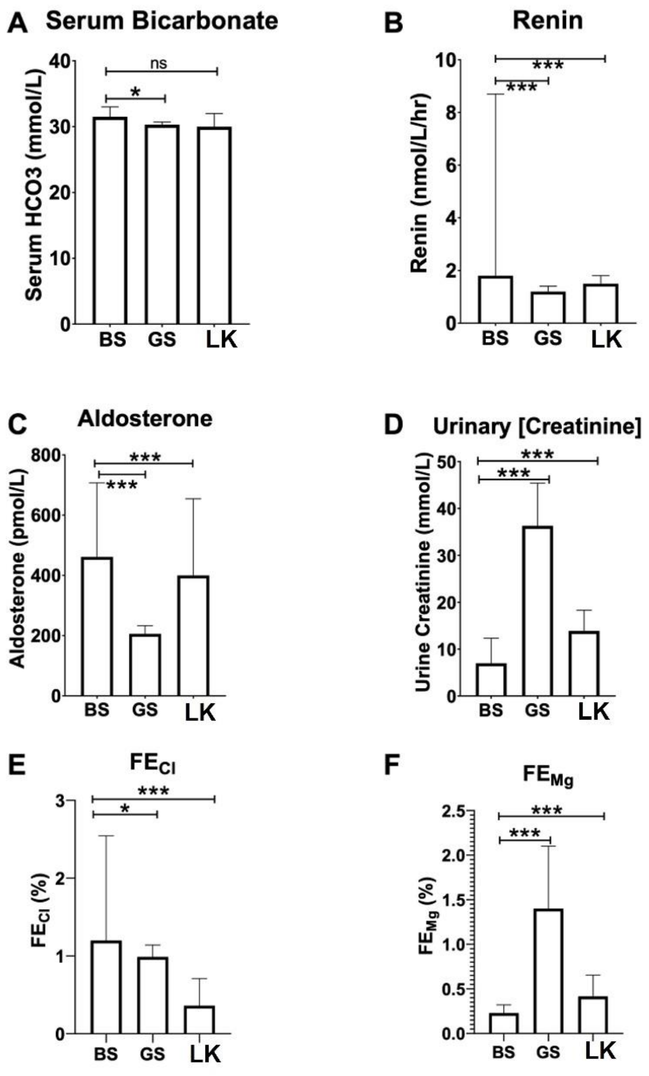

Supplementary figure 1

Figure S1. Significant biochemical differences between type 3 Bartter syndrome, Gitelman syndrome and other hypokalemic patients in the combined cohort. Data expressed as medians with 95% confidence interval. Significance is determined by the Mann-Whitney test (between 2 groups) or the Krukshal-Wallis test (between all groups). ns, nonsignificant; $*P < 0.05$; $***P < 0.001$; $****P < 0.0001$.

Table S1: Bivariate analysis of the London cohort, other hypokalaemic (LK) vs. Salt-losing tubulopathy (SLT) patients. Values displayed are median with interquartile range. Test shows which statistical test was used to determine significance, Mann Whitney (MW) for non-normally distributed data or the student's T test (T-test) for normally distributed data.

	LK (n=27)	SLT (n=39)	Test	P-value	Diff in means (95% CI)
Age (years)	42 (29-51)	39 (28-45)	T-test	0.05087	7.68 (-15.4-0.30)
Serum sodium (mmol/L)	140 (139-141)	141 (138-142)	T-test	0.748	0.276 (-1.43-1.99)
Serum potassium (mmol/L)	3.7 (3.4-4.1)	3.2 (2.8-3.7)	T-test	0.00479*	0.49 (-0.817 - -0.153)
Serum urea (mmol/L)	6.082 (3.725-7)	6.113 (3.9-7.225)	T-test	0.319	0.91 (-0.90-2.73)
Serum creatinine	95.35 (72.25-110)	76.47 (55.75-85.5)	MW	0.6455	
Serum urate (mmol/L)	0.3110 (0.2380-0.3900)	0.271 (0.230-0.370)	MW	0.4965	
Serum bicarbonate (mmol/L)	28.85 (25-32)	29.77 (27-31.75)	T-test	0.41	1.10 (-1.62-3.85)
Serum chloride (mmol/L)	97.9 (93.5-103)	94.7 (92.5-98)	T-test	0.20	2.17 (-5.43-1.18)
Urine calcium (mmol/L)	1.800 (0.925-3.600)	1.100 (0.50-2.50)	MW	0.1468	
Serum corrected calcium (mmol/L)	2.392 (2.305-2.48)	2.406 (2.312-2.5)	T-test	0.29	0.038 (-0.034-0.11)
Serum phosphate (mmol/L)	1.168 (1.03-1.31)	1.083 (1.002-1.188)	T-test	0.24	0.67 (-0.18-0.046)

Serum magnesium (mmol/L)	0.751 (0.638-0.853)	0.662 (0.563-0.74)	T-test	0.45	0.033 (-0.12-0.055)
Renin (nmol/L/hr)	5.07 (1.76-5.26)	11.71 (2.79-18.21)	MW	0.00318*	
Serum aldosterone (pmol/L)	827 (242-1117)	870 (210-930)	MW	0.1868	
Urine sodium (mmol/L)	68.1 (26.5-111)	73.8 (36.8-91.3)	MW	0.7263	
Urine potassium (mmol/L)	74.72 (25-107.75)	64.29 (45-78.75)	MW	0.1416	
Urine creatinine (µmol/L)	8380 (4305-11985)	6000 (4045-7685)	MW	0.00032*	
Urine urea (mmol/L)	198 (124-300)	134 (83-175)	MW	0.1971	
Urine chloride (mmol/L)	46 (31.5-100.5)	64.5 (41.5-108.25)	MW	0.3576	
Urine magnesium (mmol/L)	3.175 (1.175-4.525)	2.095 (1.25-2.475)	MW	0.02202*	
Systolic blood pressure (mmHg)	125 (113-134)	116.9 (108.5-120)	MW	0.4065	
Diastolic blood pressure (mmHg)	78 (73-83)	72.58 (70.5-74)	T-test	0.26	5.42 (-15.68-4.79)
Weight (kg)	78.0 (58.3-83)	66.34 (56.7-79.4)	T-test	0.78	2.73 (-22.4-16.9)
Fractional excretion of chloride (%)	2.193 (1.145-3.283)	2.353 (1.538-2.933)	T-test	0.00069*	0.73 (0.33-1.13)
Fractional excretion of sodium (%)	0.587 (0.140-0.824)	0.712 (0.387-1.00)	T-test	0.0035*	0.31 (0.11-0.52)
Fractional excretion of magnesium (%)	4.92 (1.93-7.06)	4.95 (2.29-9.12)	MW	0.1645	

Table S2: Decision table/stump rules

Simple rules used by the two most accurate classification algorithms to classify patients to a diagnosis of SLT or LK in the testing set. A) Rules for the Decision Table algorithm (predictive accuracy 75%) B) Rule for the Decision Stump algorithm (predictive accuracy 61%).

A

Serum HCO3 (mmol/L)	Fe_{Na} (%)	Diagnosis
<25.5	>0.251	LK
25.5-38.5	>0.251	SLT
>38.5	>0.251	LK
<25.5	<0.251	LK
25.5-38.5	<0.251	LK
>38.5	<0.251	LK

B

FE_{Cl} (%)	Diagnosis
<0.47966	LK
>=0.47966	SLT

Table S3: Comparison of the two best performing algorithms identified by ML (shaded columns) compared to the two best single biochemical variables identified by standard statistics in the London and combined cohorts (clear columns). Measures of accuracy include the F-measure, Receiver Operator Characteristic area under the curve (ROC area) and Precision Recall Curve area under the curve (PRC area).

	Decision Table (FE_{Na}/HCO_3)	Decision Stump (FE_{Cl})	Decision Stump (FE_{Mg})	Decision Stump (Serum Creatinine)	Decision Stump (Renin)	Decision Stump (Urinary Creatinine)
Training set						
Predictive accuracy	77%	66%	64%	73%	58%	63%
F-measure	0.77	0.65	0.6	0.75	0.57	0.6
ROC AUC	0.79	0.69	0.58	0.7	0.52	0.6
PRC AUC	0.74	0.67	0.58	0.75	0.53	0.62
Testing set						
Predictive accuracy	75%	61%	47%	52%	31%	53%
F-measure	0.73	0.62	0.52	0.49	0.2	0.57
ROC AUC	0.55	0.54	0.37	0.51	0.53	0.48
PRC AUC	0.7	0.7	0.64	0.51	0.67	0.66
Rules						
Predict WT	See Supp Table 3	<0.48%	<0.52%	>54.5	<=10.4	>16.06
Predict SLT		>=0.48%	>=0.52%	<=54.5	>10.4	<=16.06

Table S4: Genotyping data from all SLT patients. The affected gene and variant are detailed in columns 2-4. If a second variant is present (i.e. a compound heterozygote), this is detailed in columns 5-6.

Cohort	Gene	Variant 1	Variant 1 (AA change)	Variant 2	Variant 2 (AA change)
London	SLC12A3	c.2891G>A	p.Asp311Glyfs*22	c.2221G>A	p.Gly741Arg;p.Arg964Gln
London	SLC12A3	c.1928C>T	p.Pro643Leu	Deletion exon 14	
London	SLC12A3	c.1664C>T	p.Ser555Leu	c.2882+1G>T	p.(?)
London	SLC12A3	c.1664C>T	p.Ser555Leu	c.2883+1G>T	p.(?)
London	SLC12A3	c.427A>G	p.Met143Val	c.2221G>A	Gly741Arg
London	SLC12A3	c.1028T>A	p.Met343Lys	c.1028T>A	p.Met343Lys
London	SLC12A3	c.1000C>T	p.Arg334Trp	c.2221G>A	p.Gly741Arg
London	SLC12A3	Deletion exons 1-7		c.1825+1del	p.Glu609Argfs*2
London	SLC12A3	c.2186G>C	p.Gly729Ala	c.2883+1G>T	p.(?)
London	SLC12A3	c.1939del	p.Val647CysfsTer25	c.283+1G>T	p.(?)
London	SLC12A3	c.1964G>A	p.Arg655His	c.2221G>A	p.Gly741Arg
London	SLC12A3	c.910A>C	p.Thr304Pro	c.2883+1G>T	del exon 24
London	SLC12A3	c.111T>A	p.Tyr37Ter		
London	SLC12A3	c.1261T>C	p.Cys421Arg	c.1930del	p.Gln644SerfsTer
London	SLC12A3	c.2221G>8	Gly741Arg	c.2965G>A	Gly989Arg
London	SLC12A3	c.237_238dup	p.Arg80ProfsTer35	c.237_238dup	Frameshift starting Arg80 with new STOP codon 34 positions downstream
London	SLC12A3	c.363G>C	p.Glu121Asp		
London	SLC12A3	c.1925G>A	p.Arg642His	c.2965G>A	Gly989Arg
London	SLC12A3	c.2368+1del	p.(?)	SLC12A3_Ex6del	
London	SLC12A3	c.2576T>C	Leu859Pro	c.2965G>A	Gly989Arg
London	SLC12A3	c.2221G>A	Gly741Arg	c.2581C>T*	Arg861Cys
London	SLC12A3	c.961C>T	p.Arg321Trp	c.2883+1G>T	p.(?)
London	SLC12A1	c.1215G>A	p.Glu405Glu possible splicing effect	c.1215G>A	p.Glu405Glu possible splicing effect
London	SLC12A1	c.1316G>A	p.Arg439Gln	c.1316G>A	p.Arg439Gln
London	CLCNKB	Duplication Ex.4_8			
London	CLCNKB	c.1897del	p.Leu633Ter	c.1897del	p.Leu633Ter
London	CLCNKB	Whole gene deletion		Whole gene deletion	
London	CLCNKB	Whole gene deletion		Whole gene deletion	

London	CLCNKB	c.887G>A	p.Gly296Asp	c.1929+1G>A	p.(?)
London	CLCNKB	c.887G>A	p.Gly296Asp	c.1929+1G>A	p.(?)
London	CLCNKB	Whole gene deletion		Whole gene deletion	
London	CLCNKB	c.610G>A	p.Ala204Thr	c.610G>A	p.Ala204Thr
London	CLCNKB	Whole gene deletion		Whole gene deletion	
London	CLCNKB	c.1897delC	p.Leu633Ter		
London	CLCNKB	c.1987A>T	p.Arg663Ter	c.1987A>T	p.Arg663Ter
London	CLCNKB	NC_000001.10:g.16383308_16383569del		g.16383308_16383569del	
London	KCNJ1	c.89G>A	p.Cys30Tyr		
London	KCNJ1	c.89G>A	p.Cys30Tyr	c.89G>A	p.Cys30Tyr
Kobe	CLCNKB	Ex1-2 deletion		c.1830G>A	p.Trp610*
Kobe	CLCNKB	Ex1-2 deletion		c.1830G>A	p.Trp610*
Kobe	CLCNKB	c.1830G>A	p.Trp610*		
Kobe	CLCNKB	c.1830G>A	p.Trp610*		
Kobe	CLCNKB	c.1830G>A	p.Trp610*		
Kobe	CLCNKB	Ex1-2 deletion		c.1830G>A	p.Trp610*
Kobe	CLCNKB	Ex1-2 deletion		c.1830G>A	p.Trp610*
Kobe	CLCNKB	c.1830G>A	p.Trp610*		
Kobe	CLCNKB	c.1334_1335delCT	p.Ser445Phefs*5	c.1830G>A	p.Trp610*
Kobe	CLCNKB	c.1172G>A	p.Trp391*		
Kobe	CLCNKB	Ex1-19 deletion		c.1830G>A	p.Trp610*
Kobe	CLCNKB	c.1172G>A	p.Trp391*		
Kobe	CLCNKB	c.1830G>A	p.Trp610*		
Kobe	CLCNKB	c.1830G>A	p.Trp610*		
Kobe	CLCNKB	Ex1-19 deletion			
Kobe	CLCNKB	c.1830G>A	p.Trp610*		
Kobe	CLCNKB	c.1297+1G>A	IVS13+1G>A	c.1830G>A	p.Trp610*
Kobe	CLCNKB	c.100+2T>C	IVS2+2T>C	c.1830G>A	p.Trp610*
Kobe	CLCNKB	c.226C>T	p.Arg76*	c.1830G>A	p.Trp610*
Kobe	CLCNKB	c.1830G>A	p.Trp610*		
Kobe	CLCNKB	c.1033G>A	p.Gly345Ser	c.1172G>A	p.Trp391*
Kobe	CLCNKB	c.1830G>A	p.Trp610*		

Kobe	CLCNKB	c.910C>T	p.Arg304*	c.1830G>A	p.Trp610*
Kobe	CLCNKB	c.655G>T	p.Gly219Cys	c.1845+1G>A	IVS17ds+1 G>A
Kobe	CLCNKB	Ex15-19 duplication		c.1830G>A	p.Trp610*
Kobe	CLCNKB	c.1468G>A	p.Glu490Lys	Ex1-19 deletion	
Kobe	CLCNKB	c.1830G>A	p.Trp610*		
Kobe	CLCNKB	c.1309G>A	p.Gly437Arg	c.1830G>A	p.Trp610*
Kobe	CLCNKB	c.1309G>A	p.Gly437Arg	c.1830G>A	p.Trp610*
Kobe	CLCNKB	c.992_993insTCTGC	p.Thr332Leufs*19	c.1172G>A	p.Trp391*
Kobe	SLC12A3	c.1924C>T	p.Arg642Cys	c.2573T>A	p.Leu858His
Kobe	SLC12A3	c.1195C>T	p.Arg399Cys	c.2573T>A	p.Leu858His
Kobe	SLC12A3	c.1257_1259dup	p.Ala420dup	c.1868T>C	p.Leu623Pro
Kobe	SLC12A3	c.2029G>A	p.Val677Met	c.2573T>A	p.Leu858His
Kobe	SLC12A3	c.788_805dup18bp	p.Thr269delinsAsnTrpArgGlyLeuGlyPro	c.1196_1202dupGTGATGC	p.Ser402*
Kobe	SLC12A3	c.1195C>T	p.Arg399Cys	c.1670-1G>T	IVS13 as G-T -1
Kobe	SLC12A3	c.788_805dup18bp	p.Thr269delinsAsnTrpArgGlyLeuGlyPro	c.3052C>T	p.Arg1018*
Kobe	SLC12A3	c.1868T>C	p.Leu623Pro	c.2503C>T	p.Gln835*
Kobe	SLC12A3	c.179C>T	p.Thr60Met	c.2029G>A	p.Val677Met
Kobe	SLC12A3	c.139delC	p.His47Thrfs*67	c.1924C>T	p.Arg642Cys
Kobe	SLC12A3	c.2221G>A	p.Gly741Arg	c.2573T>A	p.Leu858His
Kobe	SLC12A3	c.2573T>A	p.Leu858His		
Kobe	SLC12A3	c.1195C>T	p.Arg399Cys	c.1924C>T	p.Arg642Cys
Kobe	SLC12A3	c.1195C>T	p.Arg399Cys	c.1924C>T	p.Arg642Cys
Kobe	SLC12A3	c.2891G>A	p.Arg964Gln		
Kobe	SLC12A3	c.179C>T	p.Thr60Met		
Kobe	SLC12A3	c.788_805dup18bp	p.Thr269delinsAsnTrpArgGlyLeuGlyPro		
Kobe	SLC12A3	c.788_805dup18bp	p.Thr269delinsAsnTrpArgGlyLeuGlyPro	c.863T>C	p.Leu288Pro
Kobe	SLC12A3	c.539C>A	p.Thr180Lys	c.1732G>A	p.Val578Met
Kobe	SLC12A3	c.2573T>A	p.Leu858His	c.2891G>A	p.Arg964Gln
Kobe	SLC12A3	c.1706C>T	p.Ala569Val	c.2573T>A	p.Leu858His
Kobe	SLC12A3	c.139delC	p.His47Thrfs*67	c.2573T>A	p.Leu858His
Kobe	SLC12A3	c.2573T>A	p.Leu858His		
Kobe	SLC12A3	c.539C>A	p.Thr180Lys	c.1868T>C	p.Leu623Pro

Kobe	SLC12A3	c.2573T>A	p.Leu858His	c.2747+2T>A	IVS23+2T>A
Kobe	SLC12A3	c.2573T>A	p.Leu858His	c.2747+2T>A	IVS23+2T>A
Kobe	SLC12A3	c.1262G>T	p.Cys421Phe		
Kobe	SLC12A3	c.539C>A	p.Thr180Lys		
Kobe	SLC12A3	c.539C>A	p.Thr180Lys		
Kobe	SLC12A3	c.817dupG	p.Ala273Glyfs*38	c.1670-191C>T	IVS13 as C-T -191
Kobe	SLC12A3	c.1868T>C	p.Leu623Pro	c.2548+253C>T	IVS21 ds C-T +253
Kobe	SLC12A3	c.179C>T	p.Thr60Met	c.2573T>A	p.Leu858His
Kobe	SLC12A3	c.1045C>T	p.Pro349Ser	c.1706C>T	p.Ala569Val
Kobe	SLC12A3	c.539C>A	p.Thr180Lys	c.3053G>A	p.Arg1018Q
Kobe	SLC12A3	c.960C>G	p.Tyr320*	c.2573T>A	p.Leu858His
Kobe	SLC12A3	c.1100C>T	p.Pro367Leu	c.2573T>A	p.Leu858His
Kobe	SLC12A3	c.1732G>A	p.Val578Met	c.2573T>A	p.Leu858His
Kobe	SLC12A3	c.1844C>T	p.Ser615Leu	c.2537_2538delTT	p.Phe846*
Kobe	SLC12A3	c.2573T>A	p.Leu858His	c.3052C>T	p.Arg1018*
Kobe	SLC12A3	c.2573T>A	p.Leu858His	c.2877_2878delAG	p.Arg959Serfs*11
Kobe	SLC12A3	c.488C>T	p.Thr163Met	c.1939delG	p.Val647Cysfs*25
Kobe	SLC12A3	c.1195C>T	p.Arg399Cys	c.2891G>A	p.Arg964Gln
Kobe	SLC12A3	c.539C>A	p.Thr180Lys	c.1195C>T	p.Arg399Cys
Kobe	SLC12A3	c.179C>T	p.Thr60Met	c.1670-1G>Ｔ	IVS13 as G-T -1
Kobe	SLC12A3	c.539C>A	p.Thr180Lys	c.805_806ins18	p.Thr269delinsAsnTrpArgGlyLeuGlyPro
Kobe	SLC12A3	c.539C>A	p.Thr180Lys	c.1924C>T	p.Arg642Cys
Kobe	SLC12A3	c.539C>A	p.Thr180Lys	c.2573T>A	p.Leu858His
Kobe	SLC12A3	c.1924C>T	p.Arg642Cys	c.2029G>A	p.Val677Met
Kobe	SLC12A3	c.2573T>A	p.Leu858His	c.2927C>T	p.Ser976Phe
Kobe	SLC12A3	c.179C>T	p.Thr60Met	c.1670-1G>Ｔ	IVS13 as G-T -1
Kobe	SLC12A3	c.488C>T	p.Thr163Met	c.2573T>A	p.Leu858His
Kobe	SLC12A3	c.179C>T	p.Thr60Met	c.3052C>T	p.Arg1018*
Kobe	SLC12A3	c.1924C>T	p.Arg642Cys	c.2573T>A	p.Leu858His
Kobe	SLC12A3	c.1706C>T	p.Ala569Val	c.2927C>T	p.Ser976Phe
Kobe	SLC12A3	c.539C>A	p.Thr180Lys		
Kobe	SLC12A3	c.1924C>T	p.Arg642Cys	c.2573T>A	p.Leu858His

Kobe	SLC12A3	c.2573T>A	p.Leu858His		
Kobe	SLC12A3	c.1868T>C	p.Leu623Pro	c.1963C>T	p.Arg655Cys
Kobe	SLC12A3	c.506-1G>A	IVS3 as G-A -1	c.2927C>T	p.Ser976Phe
Kobe	SLC12A3	c.539C>A	p.Thr180Lys	c.1924C>T	p.Arg642Cys
Kobe	SLC12A3	c.1216A>C	p.Asn406His	c.2927C>T	p.Ser976Phe
Kobe	SLC12A3	c.817dupG	p.Ala273Glyfs*38	c.1924C>T	p.Arg642Cys
Kobe	SLC12A3	c.1868T>C	p.Leu623Pro	c.1930delC	p.Gln644Serfs*28
Kobe	SLC12A3	c.1868T>C	p.Leu623Pro	c.1930delC	p.Gln644Serfs*28
Kobe	SLC12A3	c.488C>T	p.Thr163Met	c.1963C>T	p.Arg655Cys
Kobe	SLC12A3	c.3052C>T	p.Arg1018*		
Kobe	SLC12A3	c.505+5g>c	IVS3 ds G-C +5	c.1868T>C	p.Leu623Pro
Kobe	SLC12A3	c.178A>G	p.Thr60Ala	c.2573T>A	p.Leu858His
Kobe	SLC12A3	c.178A>G	p.Thr60Ala	c.2573T>A	p.Leu858His
Kobe	SLC12A3	c.664_666delATT	p.Ile222del	c.2573T>A	p.Leu858His
Kobe	SLC12A3	c.1924C>T	p.Arg642Cys	c.2573T>A	p.Leu858His
Kobe	SLC12A3	c.2537_2538delTT	p.Phe846*	c.2573T>A	p.Leu858His
Kobe	SLC12A3	c.2537_2538delTT	p.Phe846*	c.2573T>A	p.Leu858His
Kobe	SLC12A3	c.1868T>C	p.Leu623Pro		
Kobe	SLC12A3	c.539C>A	p.Thr180Lys	c.2573T>A	p.Leu858His
Kobe	SLC12A3	c.1924C>T	p.Arg642Cys	c.1963C>T	p.Arg655Cys
Kobe	SLC12A3	c.1924C>T	p.Arg642Cys	c.1963C>T	p.Arg655Cys
Kobe	SLC12A3	c.1924C>T	p.Arg642Cys	c.2573T>A	p.Leu858His
Kobe	SLC12A3	c.1930delC	p.Gln644Serfs*28	c.2573T>A	p.Leu858His
Kobe	SLC12A3	c.539C>A	p.Thr180Lys		
Kobe	SLC12A3	c.1924C>T	p.Arg642Cys	c.1930delC	p.Gln644Serfs*28
Kobe	SLC12A3	c.539C>A	p.Thr180Lys	c.1049C>T	p.Ser350Leu
Kobe	SLC12A3	c.1963C>T	p.Arg655Cys	c.2927C>T	p.Ser976Phe
Kobe	SLC12A3	c.1664C>T	p.Ser555Leu		
Kobe	SLC12A3	c.539C>A	p.Thr180Lys	c.668delT	p.Phe223Serfs*79
Kobe	SLC12A3	c.1670-1G>T	IVS13G>T	c.2573T>A	p.Leu858His
Kobe	SLC12A3	c.1844C>T	p.Ser615Leu		
Kobe	SLC12A3	c.179C>T	p.Thr60Met	c.2927C>T	p.Ser976Phe

Kobe	SLC12A3	c.788_805dup18bp	p.Thr269delinsAsnTrpArgGlyLeuGlyPro	c.3052C>T	p.Arg1018*
Kobe	SLC12A3	c.788_805dup18bp	p.Thr269delinsAsnTrpArgGlyLeuGlyPro	c.3052C>T	p.Arg1018*
Kobe	SLC12A3	c.817dupG	p.Ala273Glyfs*38	c.3052C>T	p.Arg1018*
Kobe	SLC12A3	c.2573T>A	p.Leu858His	c.3052C>T	p.Arg1018*
Kobe	SLC12A3	c.2573T>A	p.Leu858His	c.3053G>A	p.R1018Q
Kobe	SLC12A3	c.664_666delATT*	p.Ile222del	c.2573T>A	p.Leu858His
Kobe	SLC12A3	c.1732G>A	p.Val578Met	c.2573T>A	p.Leu858His
Kobe	SLC12A3	c.1A>T	p.Met1Leu	c.1868T>C p.L623P	p.Leu623Pro
Kobe	SLC12A3	c.2573T>A	p.Leu858His	c.3052C>T	p.R1018*
Kobe	SLC12A3	c.1924C>T	p.Arg642Cys	c.1930delC	p.Gln644Serfs*28
Kobe	SLC12A3	c.1278C>A*	p.Asn426Lys	c.1930delC	p.Gln644Serfs*28
Kobe	SLC12A3	c.488C>T	p.Thr163Met	c.2573T>A	p.Leu858His
Kobe	SLC12A3	c.1844C>T	p.Ser615Leu	c.1930delC	p.Gln644Serfs*28
Kobe	SLC12A3	c.788_805dup18bp	p.Thr269delinsAsnTrpArgGlyLeuGlyPro	c.1868T>C	p.Leu623Pro
Kobe	SLC12A3	c.2891G>A	p.Arg964Gln		
Kobe	SLC12A3	c.2573T>A	p.Leu858His		
Kobe	SLC12A3	c.2573T>A	p.Leu858His		
Kobe	SLC12A3	c.2573T>A	p.Leu858His	c.2891G>A	p.Arg964Gln
Kobe	SLC12A3	c.539C>A	p.Thr180Lys	c.2573T>A	p.Leu858His
Kobe	SLC12A3	c.1289G>A	p.Cys430Tyr	c.2573T>A	p.Leu858His
Kobe	SLC12A3	c.788_805dup18bp	p.Thr269delinsAsnTrpArgGlyLeuGlyPro		
Kobe	SLC12A3	c.788_805dup18bp	p.Thr269delinsAsnTrpArgGlyLeuGlyPro		
Kobe	SLC12A3	c.788_805dup18bp	p.Thr269delinsAsnTrpArgGlyLeuGlyPro		
Kobe	SLC12A3	c.247C>T	p.Arg83Trp	c.2573T>A	p.Leu858His
Kobe	SLC12A3	c.2537_2538delTT	p.Phe846*	c.2573T>A	p.Leu858His
Kobe	SLC12A3	c.2029G>A	p.Val677Met	c.2573T>A	p.Leu858His
Kobe	SLC12A3	c.2573T>A	p.Leu858His	c.3052C>T	p.R1018*
Kobe	SLC12A3	c.2573T>A	p.Leu858His	c.2686C>T*	p.Arg896*
Kobe	SLC12A3	c.1924C>T	p.Arg642Cys	c.2573T>A	p.Leu858His
Kobe	SLC12A3	c.3052C>T	p.Arg1018*		
Kobe	SLC12A3	c.626G>C*	p.Arg209Pro	c.2029G>A	p.Val677Met
Kobe	SLC12A3	c.1925G>A	p.Arg642His	c.3052C>T	p.Arg1018*

Kobe	SLC12A3	c.2573T>A	p.Leu858His	c.2891G>A	p.Arg964Gln
Kobe	SLC12A3	c.788_805dup18bp	p.Thr269delinsAsnTrpArgGlyLeuGlyPro		
Kobe	SLC12A3	c.539C>A	p.Thr180Lys	c.2573T>A	p.Leu858His
Kobe	SLC12A3	c.1732G>A	p.Val578Met	c.2573T>A	p.Leu858His
Kobe	SLC12A3	c.1271G>A*	p.Gly424Asp		
Kobe	SLC12A3	c.704C>T*	p.Thr235Met	c.1709C>T	p.Ala570Val
Kobe	SLC12A3	c1201_1210delCCTCTGGGG	p.Ala401_Gly403del	c.2891G>A	p.Arg964Gln
Kobe	SLC12A3	c.911C>T	p.Thr304Met	c.2573T>A	p.Leu858His
Kobe	SLC12A3	c.788_805dup18bp	p.Thr269delinsAsnTrpArgGlyLeuGlyPro	c.1289G>A	p.Cys430Tyr
Kobe	SLC12A3	c.788_805dup18bp	p.Thr269delinsAsnTrpArgGlyLeuGlyPro	c.1289G>A	p.Cys430Tyr
Kobe	SLC12A3	c.788_805dup18bp	p.Thr269delinsAsnTrpArgGlyLeuGlyPro	c.1826-1G>A	IVS14-1G>A
Kobe	SLC12A3	c.2573T>A	p.Leu858His	c.3052C>T	p.Arg1018*
Kobe	SLC12A3	c.1456G>A	p.Asp486Asn	c.1868T>C	p.Leu623Pro
Kobe	SLC12A3	c.1924C>T	p.Arg642Cys	c.2686C>T	p.Arg896*
Kobe	SLC12A3	c.1924C>T	p.Arg642Cys	c.2573T>A	p.Leu858His
Kobe	SLC12A3	c.1A>T	p.Met1Leu	c.179C>T	p.Thr60Met
Kobe	SLC12A3	c.2537_2538delTT	p.Phe846*	c.1923C>G	p.Tyr641*
Kobe	SLC12A3	c.1077C>G	p.Asn359Lys	c.1709C>T	p.Ala570Val
Kobe	SLC12A3	c.1868T>C	p.Leu623Pro	c.2927C>T	p.Ser976Phe
Kobe	SLC12A3	c.2891G>A	p.Arg964Gln	ex9-18deletion	
Kobe	SLC12A3	c.2573T>A	p.Leu858His	ex7-8deletion	
Kobe	SLC12A3	c.1924C>T	p.Arg642Cys	c.3053G>A	p.Arg1018Q
Kobe	SLC12A3	c.1930delC	p.Gln644Serfs*28	c.2573T>A	p.Leu858His
Kobe	SLC12A3	c.1930delC	p.Gln644Serfs*28		
Kobe	SLC12A3	c.1963C>T	p.Arg655Cys	c.2573T>A	p.Leu858His
Kobe	SLC12A3	c1924C>T	p.Arg642Cys	c.2573T>A	p.Leu858His
Kobe	SLC12A3	c.539C>A	p.Thr180Lys	c.1698C>A	p.Asn566Lys
Kobe	SLC12A3	c179C>T	p.Thr60Met	c.2099T>C	p.Leu700pro
Kobe	SLC12A3	c.1924C>T	p.Arg642Cys	c.2573T>A	p.Leu858His
Kobe	SLC12A3	c.238delC	p.(Arg80Glyfs*34)	c.2573T>A	p.Leu858His
Kobe	SLC12A3	c.2573T>A	p.Leu858His	c.2891G>A	p.Arg964Gln
Kobe	SLC12A3	c.539C>A	p.Thr180Lys	c.1868T>C	p.Leu623Pro

Kobe	SLC12A3	c.1669+297T>G	P.(?)	c.2927C>T	p.Ser976Phe
Kobe	SLC12A3	c.1897dupG	p.E633Gfs56	c.1924C>T	p.Arg642Cys
Kobe	SLC12A3	c.2191G>A	p.Gly731Arg		
Kobe	SLC12A3	c.1456G>A	p.Asp486Asn	c.2927C>T	p.Ser976Phe
Kobe	SLC12A3	c.179C>T	p.Thr60met	c.2573T>A	p.Leu858His
Kobe	SLC12A3	c.1100C>T	p.Pro367Leu	c.1924C>T	p.Arg642Cys
Kobe	SLC12A3	c.2573T>A	p.Leu858His	c.2573T>A	p.Leu858His
Kobe	SLC12A3	c.539C>A	p.Thr180Lys	c.2573T>A	p.Leu858His
Kobe	SLC12A3	c.539C>A	p.Thr180Lys	c.1868T>C	p.Leu623Pro
Kobe	SLC12A3	c.539C>A	p.Thr180Lys	c.2891G>A	p.Arg964Gln
Kobe	SLC12A3	c.2573T>A	p.Leu858His	c.2573T>A	p.Leu858His
Kobe	SLC12A3	c.2573T>A	p.Leu858His	c.3052C>T	p.Arg1018*
Kobe	SLC12A3	c.1924C>T	p.Leu642Cys	c.2573T>A	p.Leu858His
Kobe	SLC12A3	c.2573T>A	p.Leu858His	c.2573T>A	p.Leu858His
Kobe	SLC12A3	c.1942C>T	p.Leu642Cys	c.2573T>A	p.Leu858His
Kobe	SLC12A3	c.2927G>T	p.Ser976Phe	c.2927G>T	p.Ser976Phe
Kobe	SLC12A3	c.2573T>A	p.Leu858His	c.2573T>A	p.Leu858His
Kobe	SLC12A3	c.1868T>C	p.Leu623Pro	c.2927C>T	p.Ser976Phe
Kobe	SLC12A3	c.1195C>T	Arg399Cys	c.2573T>A	p.Leu858His
Kobe	SLC12A3	c.2573T>A	p.Leu858His	c.3053G>A	p.Arg1018Gln
Kobe	SLC12A3	c.788_805dup18bp	p.Thr269delinsAsnTrpArgGlyLeuGlyPro	c.1132G>A	p.Ala378Thr
Kobe	SLC12A3	c.1100C>T	p.Pro367Leu	c.1942C>T	p.Leu642Cys
Kobe	SLC12A3	c.2573T>A	p.Leu858His		
Kobe	SLC12A3	c.1163C>A	p.Ala388Asp	c.2573T>A	p.Leu858His
Kobe	SLC12A3	c.139delC	p.His47Thrfs*67	c.2660+1G>A	
Kobe	SLC12A3	c.1163C>A	p.Ala388Asp	c.2573T>A	p.Leu858His
Kobe	SLC12A3	c.539C>A	p.Thr180Lys	c.788_805dup18bp	p.Thr269delinsAsnTrpArgGlyLeuGlyPro
Taipei	SLC12A3	c.1670-191C>T	238bp-containing pseudo-exon	c1670-191C>T	238bp-containing pseudo-exon
Taipei	SLC12A3	c.268C>T	p.His90Tyr	c.268C>T	p.His90Tyr
Taipei	SLC12A3	c.37G>C/c.179C>T	p.Ala13Pro/p.Thr60Met	c.248G>A	p.Arg83Gln
Taipei	SLC12A3	c.2129C>A	p.Ser710X	c.2875-2876delAG	p.Arg959fs
Taipei	SLC12A3	c.248G>A	p.Arg83Gln	c.1670-191C>T	238bp-containing pseudo-exon

Taipei	SLC12A3	c.911C>T	p.Thr304Met	c.2875-2876delAG	p.Arg959fs
Taipei	SLC12A3	c.1000C>T	p.Arg334Trp	c.1326C>G	p.Asn442Lys
Taipei	SLC12A3	c.2875-2876delAG	p.Arg959fs		
Taipei	SLC12A3	c.179C>T	p.Thr60Met	c.179C>T	p.Thr60Met
Taipei	SLC12A3	c.2875-2876delAG	p.Arg959fs	c.2875-2876delAG	p.Arg959fs
Taipei	SLC12A3	c.2875-2876delAG	p.Arg959fs	c.2875-2876delAG	p.Arg959fs
Taipei	SLC12A3	c.185A>G / c.248G>A	p.Asp62Gly/ p.Arg83Gln	c.1541C>T	p.Ala514Val
Taipei	SLC12A3	c.179C>T	p.Thr60Met	c.2573T>A	p.Leu858His
Taipei	SLC12A3	c.2542G>A	p.Asp848Asn		
Taipei	SLC12A3	c.2542G>A	p.Asp848Asn		
Taipei	SLC12A3	c.1946C>T	p.Thr649Met		
Taipei	SLC12A3	c.1946C>T	p.Thr649Met		
Taipei	SLC12A3	IVS7-1G>A (correct: c.577-1G>A)+c.965-976 GCGGACATTTTTGT> ACCGAAAATTTT	p.(?); probably Exon7 and 8 skipping	c.1924C>T	p.Arg642Cys
Taipei	SLC12A3	c.1670-191C>T	238bp-containing pseudo-exon	Ex23 deletion	
Taipei	SLC12A3	c.488C>T /c.2612G>A	p.Thr163Met/p.Arg871His	c.2929C>A	p.Arg977X
Taipei	SLC12A3	c.488C>T /c.2612G>A	p.Thr163Met/p.Arg871His	c.2929C>A	p.Arg977X
Taipei	SLC12A3	c.1670-191C>T	238bp-containing pseudo-exon	c.1670-191C>T	238bp-containing pseudo-exon
Taipei	SLC12A3	c.1670-191C>T	238bp-containing pseudo-exon	c.1670-191C>T	238bp-containing pseudo-exon
Taipei	SLC12A3	c.2532G>A	p.Trp844X	c.2875-2876delAG	p.Arg959fs
Taipei	SLC12A3	c.488C>T /c.2612G>A	p.Thr163Met/p.Arg871His	c.2129C>A	p.Ser710X
Taipei	SLC12A3	c.488C>T /c.2612G>A	p.Thr163Met/p.Arg871His	c.2129C>A	p.Ser710X
Taipei	SLC12A3	c.2129C>A	p.Ser710X	c.2548+253C>T	90bp-containing pseudo-exon
Taipei	SLC12A3	IVS7-1G>A+c.965-976 GCGGACATTTTTGT> ACCGAAAATTTT	Exon7 and 8 skipping	c.2875-2876delAG	p.Arg959fs
Taipei	SLC12A3	c.179C>T	p.Thr60Met	c.185A>G	p.Asp62Gly
Taipei	SLC12A3	c.2129C>A	p.Ser710X		
Taipei	SLC12A3	c.2129C>A	p.Ser710X	c.2875-2876delAG	p.Arg959fs
Taipei	SLC12A3	c.488C>T /c.2612G>A	p.Thr163Met/p.Arg871His	c.1278C>A	p.Asn426Lys
Taipei	SLC12A3	c.488C>T /c.2612G>A	p.Thr163Met/p.Arg871His	c.2548+253C>T	90bp-containing pseudo-exon
Taipei	SLC12A3	c.1967C>T	p.P656L		

Taipei	CLCNKb	c.752delG	p.L252Sfs	Ex1-19deletion	
Taipei	CLCNKb	c.1004T>C	p.Lys335Pro	c.1409G>A	p.Gly470Glu
Taipei	SLC12A1	c.825T>A/c.1493C>T	p.Tyr275X/p.Ala498Val	c.2996G>A	p.Arg999His

STROBE Statement—Checklist of items that should be included in reports of ***cohort studies***

	Item No	Recommendation	Page No
**Title and abstract**	1	(*a*) Indicate the study's design with a commonly used term in the title or the abstract	2
		(*b*) Provide in the abstract an informative and balanced summary of what was done and what was found	2
**Introduction**			
Background/rationale	2	Explain the scientific background and rationale for the investigation being reported	3
Objectives	3	State specific objectives, including any prespecified hypotheses	3
**Methods**			
Study design	4	Present key elements of study design early in the paper	5
Setting	5	Describe the setting, locations, and relevant dates, including periods of recruitment, exposure, follow-up, and data collection	5
Participants	6	(*a*) Give the eligibility criteria, and the sources and methods of selection of participants. Describe methods of follow-up	5
		(*b*) For matched studies, give matching criteria and number of exposed and unexposed	N/A
Variables	7	Clearly define all outcomes, exposures, predictors, potential confounders, and effect modifiers. Give diagnostic criteria, if applicable	5
Data sources/ measurement	8*	For each variable of interest, give sources of data and details of methods of assessment (measurement). Describe comparability of assessment methods if there is more than one group	5<br>6

Bias	9	Describe any efforts to address potential sources of bias	6
Study size	10	Explain how the study size was arrived at	5
Quantitative variables	11	Explain how quantitative variables were handled in the analyses. If applicable, describe which groupings were chosen and why	5
Statistical methods	12	(*a*) Describe all statistical methods, including those used to control for confounding	5-6
		(*b*) Describe any methods used to examine subgroups and interactions	6
		(*c*) Explain how missing data were addressed	6
		(*d*) If applicable, explain how loss to follow-up was addressed	N/A
		(*e*) Describe any sensitivity analyses	7
**Results**			
Participants	13*	(a) Report numbers of individuals at each stage of study—eg numbers potentially eligible, examined for eligibility, confirmed eligible, included in the study, completing follow-up, and analysed	8
		(b) Give reasons for non-participation at each stage	N/A – single timepoint
		(c) Consider use of a flow diagram	
Descriptive data	14*	(a) Give characteristics of study participants (eg demographic, clinical, social) and information on exposures and potential confounders	8
		(b) Indicate number of participants with missing data for each variable of interest	
		(c) Summarise follow-up time (eg, average and total amount)	N/A
Outcome data	15*	Report numbers of outcome events or summary measures over time	

Main results	16	(*a*) Give unadjusted estimates and, if applicable, confounder-adjusted estimates and their precision (eg, 95% confidence interval). Make clear which confounders were adjusted for and why they were included<br>(*b*) Report category boundaries when continuous variables were categorized<br>(*c*) If relevant, consider translating estimates of relative risk into absolute risk for a meaningful time period	8
Other analyses	17	Report other analyses done—eg analyses of subgroups and interactions, and sensitivity analyses	10
**Discussion**			
Key results	18	Summarise key results with reference to study objectives	11
Limitations	19	Discuss limitations of the study, taking into account sources of potential bias or imprecision. Discuss both direction and magnitude of any potential bias	11,13
Interpretation	20	Give a cautious overall interpretation of results considering objectives, limitations, multiplicity of analyses, results from similar studies, and other relevant evidence	11
Generalisability	21	Discuss the generalisability (external validity) of the study results	25
**Other information**			
Funding	22	Give the source of funding and the role of the funders for the present study and, if applicable, for the original study on which the present article is based	16

*Give information separately for exposed and unexposed groups.

Note: An Explanation and Elaboration article discusses each checklist item and gives methodological background and published examples of transparent reporting. The STROBE checklist is best used in conjunction with this article (freely available on the Web sites of PLoS Medicine at http://www.plosmedicine.org/, Annals of Internal Medicine at http://www.annals.org/, and Epidemiology at http://www.epidem.com/). Information on the STROBE Initiative is available at http://www.strobe-statement.org.
